# Whole Genome Sequencing of Extended-Spectrum- and AmpC- β-Lactamase-Positive Enterobacterales Isolated From Spinach Production in Gauteng Province, South Africa

**DOI:** 10.3389/fmicb.2021.734649

**Published:** 2021-10-01

**Authors:** Loandi Richter, Erika M. du Plessis, Stacey Duvenage, Mushal Allam, Arshad Ismail, Lise Korsten

**Affiliations:** ^1^Department of Plant and Soil Sciences, University of Pretoria, Pretoria, South Africa; ^2^Department of Science and Innovation, National Research Foundation Centre of Excellence in Food Security, Pretoria, South Africa; ^3^Sequencing Core Facility, National Institute for Communicable Diseases, National Health Laboratory Service, Johannesburg, South Africa

**Keywords:** WGS, food safety, leafy greens, multidrug resistance, foodborne bacterial pathogens

## Abstract

The increasing occurrence of multidrug-resistant (MDR) extended-spectrum β-lactamase- (ESBL) and/or AmpC β-lactamase- (AmpC) producing Enterobacterales in irrigation water and associated irrigated fresh produce represents risks related to the environment, food safety, and public health. In South Africa, information about the presence of ESBL/AmpC-producing Enterobacterales from non-clinical sources is limited, particularly in the water–plant-food interface. This study aimed to characterize 19 selected MDR ESBL/AmpC-producing *Escherichia coli* (*n*=3), *Klebsiella pneumoniae* (*n*=5), *Serratia fonticola* (*n*=10), and *Salmonella enterica* (*n*=1) isolates from spinach and associated irrigation water samples from two commercial spinach production systems within South Africa, using whole genome sequencing (WGS). Antibiotic resistance genes potentially encoding resistance to eight different classes were present, with *bla*_CTX-M-15_ being the dominant ESBL encoding gene and *bla*_ACT_-types being the dominant AmpC encoding gene detected. A greater number of resistance genes across more antibiotic classes were seen in all the *K. pneumoniae* strains, compared to the other genera tested. From one farm, *bla*_CTX-M-15_-positive *K. pneumoniae* strains of the same sequence type 985 (ST 985) were present in spinach at harvest and retail samples after processing, suggesting successful persistence of these MDR strains. In addition, ESBL-producing *K. pneumoniae* ST15, an emerging high-risk clone causing nosocomical outbreaks worldwide, was isolated from irrigation water. Known resistance plasmid replicon types of Enterobacterales including IncFIB, IncFIA, IncFII, IncB/O, and IncHI1B were observed in all strains following analysis with PlasmidFinder. However, *bla*_CTX-M-15_ was the only β-lactamase resistance gene associated with plasmids (IncFII and IncFIB) in *K. pneumoniae* (*n*=4) strains. In one *E. coli* and five *K. pneumoniae* strains, integron In191 was observed. Relevant similarities to human pathogens were predicted with PathogenFinder for all 19 strains, with a confidence of 0.635–0.721 in *S. fonticola*, 0.852–0.931 in *E. coli*, 0.796–0.899 in *K. pneumoniae*, and 0.939 in the *S. enterica* strain. The presence of MDR ESBL/AmpC-producing *E. coli*, *K. pneumoniae*, *S. fonticola*, and *S. enterica* with similarities to human pathogens in the agricultural production systems reflects environmental and food contamination mediated by anthropogenic activities, contributing to the spread of antibiotic resistance genes.

## Introduction

The discovery of antibiotics in the 1940s led to a new age in medical care. However, the global increase in antimicrobial resistance (AMR) is reducing the effectiveness of clinically important antibiotics (Lobanovska and Pilla, 2017; Dandachi et al., 2019). Examples of shifting resistance profiles in bacteria within the β-lactam class of antibiotics, including penicillins and third generation cephalosporins, which are the most widely used in human and veterinary medicine and widely expressed AMR are being reported (Finton et al., 2020). Persistent exposure to these antibiotics has resulted in bacteria becoming resistant by evolving extended-spectrum β-lactamases (ESBLs), which hydrolyze the β-lactam ring within the antibiotic, thus rendering it inactive (Bush and Jacoby, 2010). Consequently, production of ESBLs is regarded as one of the most clinically significant resistance mechanisms (Bush and Jacoby, 2010), with ESBL-producing Enterobacterales (*Escherichia coli*, *Klebsiella pneumoniae*, and *Serratia* spp., among others) listed as priority pathogens for research and development in the new frontier of antibiotics (WHO, 2017).

Classified into several groups according to their amino acid sequence homology, the CTX-M, TEM, and SHV ESBL variants are the most common β-lactamases identified in Enterobacterales (van Duin and Doi, 2017). In addition, AmpC β-lactamases (AmpCs) are chromosomally encoded by several Enterobacterales species and play a key role in resistance development (van Duin and Doi, 2017). Plasmid encoded AmpC genes have been known since 1989 (Jacoby, 2009) and are now regularly reported in clinical and environmental strains (Khari et al., 2016; Colosi et al., 2020; Tekele et al., 2020). Both chromosomally encoded and plasmid-mediated AmpC β-lactamases confer resistance to a broad spectrum of β-lactams such as penicillins, oxyimino-cephalosporins (including cefotaxime and ceftazidime), cephamycins, and aztreonam at variable levels (Jacoby, 2009; Palzkill, 2018; Furlan and Stehling, 2021; Lopes et al., 2021b).

The increase in antimicrobial resistant strains and effective resistance mechanisms among Enterobacterales has led to numerous global reports of ESBLs, AmpC-, and more recently carbapenemase-producing Enterobacterales not only in clinical settings, but also in the agricultural environment (Ye et al., 2017; Al-Kharousi et al., 2019; Dandachi et al., 2019; Hassen et al., 2020; Richter et al., 2020). Although members of the Enterobacterales family occur naturally in human and animals’ gastrointestinal tracts as well as in the environment (water, soil, and plants; Blaak et al., 2014; Ye et al., 2017), the occurrence of multidrug-resistant (MDR) strains in the different habitats is concerning. Inadequately treated or untreated effluents from industries, households, and zootechnical farms are reported as one of the main contamination causes of South African surface- and ground water resources (Verlicchi and Grillini, 2020). It is also well-documented that the three principal antibiotic contamination channels in the environment are animal-, human-, and manufacturing waste (O’neill, 2016). Consequently, contamination of soil, irrigation-, and drinking water as well as crops can occur, adding additional exposure routes to humans (Finton et al., 2020; Lopes et al., 2021a).

Previous surveillance studies have shown prevalence of MDR ESBL/AmpC-producing Enterobacterales in fresh vegetables sold in South Africa (Richter et al., 2019) and in other countries e.g., the Netherlands, Switzerland, and Germany (Reuland et al., 2014; Zurfluh et al., 2015; Reid et al., 2020). Occurrence of ESBL-producing Enterobacterales has also been reported in corresponding irrigation water sources and cultivated crops (Blaak et al., 2014; Njage and Buys, 2014; Ye et al., 2017). Furthermore, Richter et al. (2020) reported the occurrence of ESBL/AmpC-producing Enterobacterales in different spinach supply chains from irrigation water and produce at harvest, throughout processing and at retail in the Gauteng Province of South Africa.

The high discriminatory power of whole genome sequencing (WGS) has led to an increase in use of this method for detecting points of contamination, source tracking, pathogen surveillance, and outbreak investigations (Oniciuc et al., 2018; CDC, 2019). WGS provides information regarding multiple AMR genes, genomic mutations, mobile genetic elements, and association with resistance genes, as well as molecular typing like multi-locus sequence typing (MLST; Oniciuc et al., 2018; CDC, 2019; Kim et al., 2020). Consequently, the WGS results can aid in elucidating the genetic relationship among isolates from different environments and along the food chain (Adator et al., 2020). Surveillance of antimicrobial resistant strains through WGS is increasingly being used due to increasing accessibility and affordability (Adator et al., 2020). In South Africa, WGS has been used for characterization of clinical ESBL-producing *K. pneumoniae* strains among others (Founou et al., 2019), as well as typing of *Listeria monocytogenes* from environmental and clinical settings during the 2017 listeriosis outbreak (Thomas et al., 2020). However, the use of WGS for surveillance of antimicrobial resistant potential pathogenic Enterobacterales in retailed fresh produce and the production environment has not been reported locally.

The World Health Organization (WHO) developed the Global AMR Surveillance System (GLASS) in 2015 supporting research and surveillance as well as a global data sharing through a standardized analysis approach (WHO, 2020). Initially, the GLASS focus was mainly on surveillance of human priority pathogens, but has since shifted to include AMR in foodborne pathogens (WHO, 2020). Moreover, the One Health framework for understanding AMR in pathogenic gram-negative bacteria is increasingly attracting attention (Collignon and McEwen, 2019). In South Africa, information regarding AMR in fresh produce production systems and specifically focusing on the Enterobacterales is lacking. The aim of this study was thus to use WGS for analysis of AMR genes, associated mobile genetic elements, virulence factors, serotypes, multi-locus sequence types, and pathogenicity of selected, partially characterized, ESBL/AmpC-producing environmental Enterobacterales from commercial spinach production systems (Richter et al., 2020). These isolates included four different species (*E. coli*, *K. pneumoniae*, *S. fonticola*, and *S. enterica*) listed by the WHO as a particular threat for gram-negative bacteria that are resistant to multiple antibiotics (WHO, 2017), while isolates harbouring integrons as described in Richter et al. (2020) were preferentially selected. The results of this study will contribute to address the problem of antimicrobial drug resistance at the water–plant-food interface and how it might impact human health and disease.

## Materials and Methods

### Isolation and DNA Extraction of ESBL/AmpC-Producing Enterobacterales

Irrigation water and fresh produce samples from spinach production systems were collected and ESBL-producing Enterobacterales were isolated as described (Richter et al., 2020). A selection of 19 isolates were further characterized (Table 1). The genomic DNA of each isolate was extracted with the DNeasy PowerSoil kit (Qiagen, South Africa) according to the manufacturer’s instructions. Following gDNA extraction, the concentrations were determined using the Qubit dsDNA Broad Range Assay and a Qubit 2.0 fluorometer (Life Technologies, Johannesburg) and quantification was determined on a Nanodrop 2000 (ThermoScientific, Johannesburg).

**Table 1:** Isolates selected for whole genome sequence analysis from the agricultural environment in spinach supply chains, Gauteng Province, South Africa.

Strain	Organism identity	Source	Isolation point from spinach production systems
		Water (W) or spinach (S)	
UPMP2117	*Escherichia coli*	W	Water reservoir
UPMP2120	*Escherichia coli*	S	Unwashed spinach bunches at retailer
UPMP2130	*Escherichia coli*	W	Holding dam water (source water)
UPMP2112	*Klebsiella pneumoniae*	W	Irrigation pivot point water
UPMP2114	*Klebsiella pneumoniae*	S	Spinach at harvest
UPMP2118	*Klebsiella pneumoniae*	W	Irrigation pivot point water
UPMP2121	*Klebsiella pneumoniae*	S	Unwashed spinach bunches at retailer
UPMP2122	*Klebsiella pneumoniae*	S	Spinach at retailer
UPMP2115	*Salmonella spp.*	W	River water
UPMP2116	*Serratia fonticola*	W	River water
UPMP2119	*Serratia fonticola*	W	Irrigation pivot point water
UPMP2123	*Serratia fonticola*	S	Unwashed spinach punnet at retailer
UPMP2124	*Serratia fonticola*	S	Spinach at receival
UPMP2125	*Serratia fonticola*	S	Spinach after pack
UPMP2126	*Serratia fonticola*	S	Spinach at receival
UPMP2127	*Serratia fonticola*	S	Unwashed spinach at retailer
UPMP2128	*Serratia fonticola*	S	Unwashed spinach at retailer
UPMP2129	*Serratia fonticola*	S	Spinach at receival
UPMP2131	*Serratia fonticola*	S	Unwashed spinach at retailer

### DNA Sequencing and Whole Genome Analysis

Sequencing was performed on an Illumina MiSeq instrument (2×300bp) with 100× coverage by the National Institute for Communicable Diseases Sequencing Core Facility, South Africa, following preparation of multiplexed paired-end libraries with the Nextera XT DNA sample preparation kit (Illumina, San Diego, CA, United States). The resultant reads were quality trimmed using CLC version 20^1^ and *de novo* assembled. The contiguous sequences were then submitted to the National Centre for Biotechnology Information (NCBI) Prokaryotic Genome Annotation Pipeline.^2^ The AMR gene presence was corroborated using ABRicate^3^ that included the Comprehensive Antibiotic Resistance Database (CARD), ARG-ANNOT, ResFinder, NCBI AMRFinder Plus, and MEGARes databases (Zankari et al., 2012; Gupta et al., 2014; Jia et al., 2017; Feldgarden et al., 2019; Doster et al., 2020). Plasmid replicon types were determined with PlasmidFinder (version 2.1; Carattoli et al., 2014). Using the Centre for Genomic Epidemiology (CGE) platform^4^, mobile genetic elements for all four species, sequence types of *E. coli*, *K. pneumoniae*, and *S. enterica* as well as the *E. coli* serotypes based on lipopolysaccharide (O-antigen) and capsular flagella (protein; H-antigen), and virulence genes of *E. coli* were determined with MGEFinder, Multilocus Sequence Typing (MLST; version 2.2), SeroTypeFinder (version 2.0), and VirulenceFinder (version 2.0), respectively (Larsen et al., 2012; Joensen et al., 2014, 2015; Johansson et al., 2021). The following parameters were used in the Serotype Finder Web-based tool: 85% threshold for %ID and 60% minimum length (the number of nucleotides in a sequence of interest that must overlap a serotype gene to count as a hit for that gene; Joensen et al., 2015). The *in silico* serotyping based on the capsule polysaccharide (K-antigen) of *K. pneumoniae* strains was conducted using Kaptive Web (Wick et al., 2018), while the presence of virulence genes for *K. pneumoniae* was identified by using the Institut Pasteur’s *Klebsiella* database.^5^ Additionally, paired reads of the WGS raw data files for the *S. enterica* strain were uploaded to the online SeroSeq tool version 1.0 which predicted the *Salmonella* serotype of the requested isolate (Zhang et al., 2015; Thompson et al., 2018). The *Salmonella* Pathogenicity Islands (SPI) were identified with SPIFinder 2.0 (Roer et al., 2016). Next, the existence of virulence factors in each SPI was analyzed by performing BLAST analysis on the predicted SPIs against the virulence factor database (VFDB; Chen et al., 2016; Ashari et al., 2019). The virulence factors of *S. fonticola* were determined using the VFDB with ABRicate (Chen et al., 2016). All sequences were submitted to the INTEGRALL database^6^ for annotation and integron number assignment. Using PathogenFinder (version 1.1) on the CGE platform, the strains’ pathogenicity towards humans was predicted (Cosentino et al., 2013).

### Data Availability

The nucleotide sequences of the 19 Enterobacterales strains described in this paper were deposited in the National Center for Biotechnology Information GenBank database in the BioProject number: PRJNA642017, accession numbers NZ_JACAAL010000000, NZ_JACBIV000000000-NZ_JACBJE000000000, and NZ_JACNYM000000000-NZ_JACNYT000000000 (Table 2).

**Table 2:** *In silico* multi-locus sequence typing (MLST) analysis, predicted serotypes, and pathogenicity probability of Enterobacterales isolated from irrigation water and spinach throughout production from farm to retail.

Accession	Strain	Source	Species	Sequence type	Serotype	Pathogenicity probability
NZ_JACNYS000000000	UPMP2120	S	*Escherichia coli*	ST58	O75:H9	0.888
NZ_JACNYT000000000	UPMP2117	W	*Escherichia coli*	ST117	O11:H4	0.931
NZ_JACNYN000000000	UPMP2130	W	*Escherichia coli*	ST10	O8:H17	0.852
NZ_JACAAL010000000	UPMP2112	W	*Klebsiella pneumoniae*	ST3559	KL27:O4	0.899
NZ_JACBJB000000000	UPMP 2118	W	*Klebsiella pneumoniae*	ST15	KL24:O1v1	0.889
NZ_JACBJE000000000	UPMP2114	S	*Klebsiella pneumoniae*	ST985	KL39:O1v2	0.885
NZ_JACBIZ000000000	UPMP2121	S	*Klebsiella pneumoniae*	ST985	KL39:O1v2	0.796
NZ_JACBIY000000000	UPMP2122	S	*Klebsiella pneumoniae*	ST985	KL39O1v1	0.885
NZ_JACBJD000000000	UPMP2115	W	*Salmonella enterica*	ST4924	Pretoria	0.939
NZ_JACBJC000000000	UPMP2116	W	*Serratia fonticola*	N.D	N.D	0.721
NZ_JACBJA000000000	UPMP2119	W	*Serratia fonticola*	N.D	N.D	0.699
NZ_JACBIX000000000	UPMP2123	S	*Serratia fonticola*	N.D	N.D	0.692
NZ_JACNYR000000000	UPMP2124	S	*Serratia fonticola*	N.D	N.D	0.635
NZ_JACNYQ000000000	UPMP2125	S	*Serratia fonticola*	N.D	N.D	0.645
NZ_JACNYP000000000	UPMP2126	S	*Serratia fonticola*	N.D	N.D	0.659
NZ_JACNYO000000000	UPMP2127	S	*Serratia fonticola*	N.D	N.D	0.659
NZ_JACBIW000000000	UPMP2128	S	*Serratia fonticola*	N.D	N.D	0.674
NZ_JACBIV000000000	UPMP2129	S	*Serratia fonticola*	N.D	N.D	0.659
NZ_JACNYM000000000	UPMP2131	S	*Serratia fonticola*	N.D	N.D	0.705

W, water; S, spinach; and N.D., not determined.

## Results

### Detection of Antimicrobial Resistance Genes

**Figure 1:**
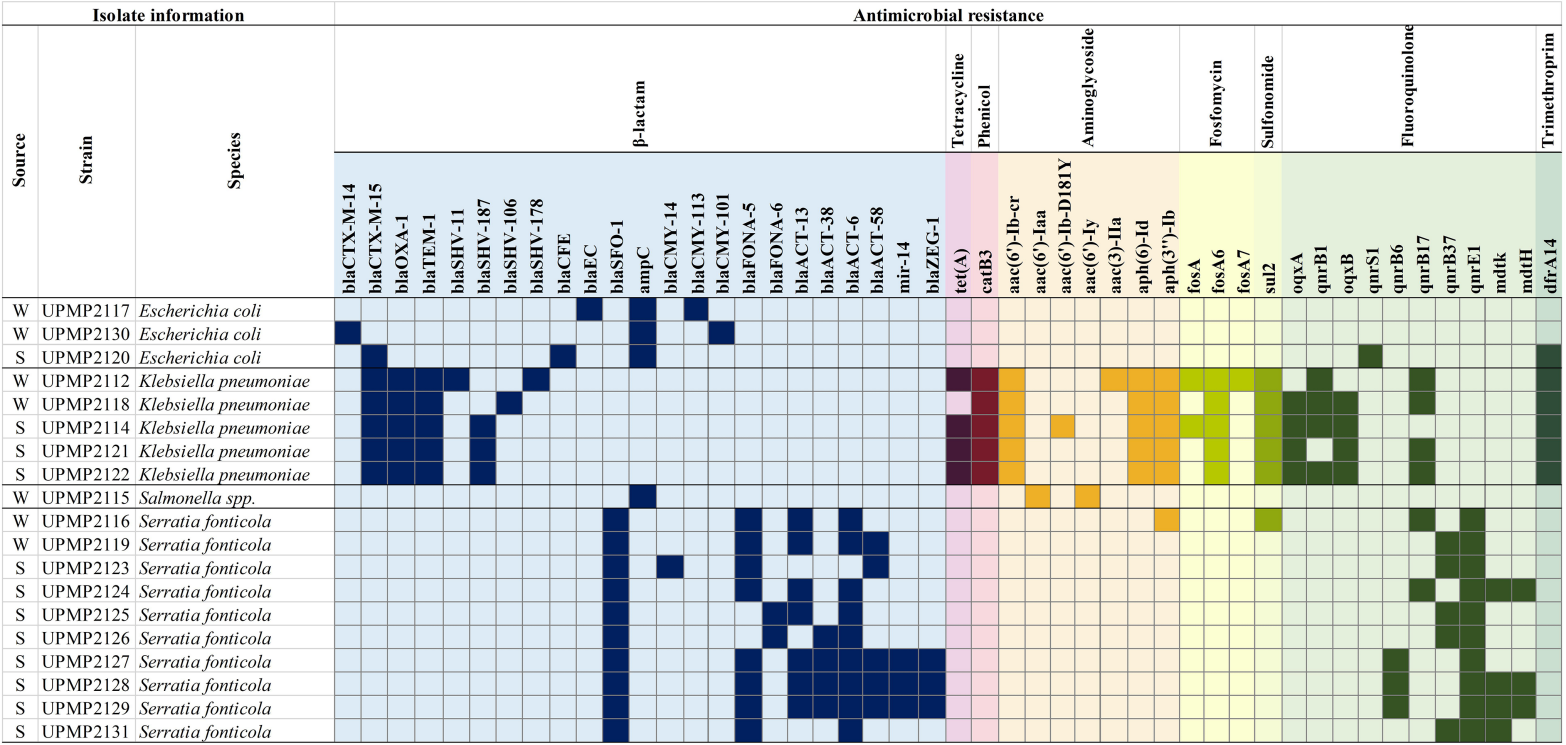
Antimicrobial resistance (AMR) genes present in Enterobacterales isolated from water and spinach from farm to retail. W, water; S, spinach.

The selected 19 ESBL/AmpC producing Enterobacterales isolates all harboured at least one β-lactamase encoding gene in addition to the ESBL/AmpC genetic determinants, accompanied by resistance genes from different antibiotic classes including fluoroquinolone, sulfonomide, fosfomycin, aminoglycoside, trimethroprim, phenicol, and/or tetracycline (Figure 1). The β-lactamase resistance genes included chromosomally encoded AmpC in the *S. enterica* strain as well as all three *E. coli* strains. Plasmid-mediated AmpC genes (*bla*_CMY-113_ and *bla*_CMY-101_) were present in two *E. coli* strains from irrigation water and *bla*_ACT-13_, *bla*_ACT-38_, *bla*_ACT-6_, and/or *bla*_ACT-58_ were present in 10 *S. fonticola* strains from irrigation water (*n*=2) and spinach (*n*=8) samples (Figure 1). Additionally, *bla*_FONA-5_ (*n*=8) from irrigation water and spinach and *bla*_FONA-6_ (*n*=2) from spinach were present in *S. fonticola* strains. The ESBL genes included *bla*_SFO-1_ in all 10 *S. fonticola* strains, *bla*_CTX-M-15_ in five *K. pneumoniae* strains from irrigation water and spinach, and one *E. coli* strain from spinach. It also included *bla*_CTX-M-14_ in an *E. coli* strain from irrigation water, while *bla*_SHV-187_ (*n*=3), *bla*_SHV-106_ (*n*=1), and *bla*_SHV-178_ (*n*=1) were present in *K. pneumoniae* strains (Figure 1).

Interestingly, a greater number of resistance genes across more classes were seen in all the *K. pneumoniae* strains (*n*=5), compared to the other genera tested. All five *K. pneumoniae* strains had chloramphenicol (*catB3*), aminoglycosides [*aac(6')-Ib-cr*, *aph(6)-Id* and *aph(3")-Ib*], fosfomycin (*fosA6*), and sulfonomide (*sul2*) resistance genes present (Figure 1). Other resistance genes included fluoroquinolone *oqxA* (*n*=4), *oqxB* (*n*=4), and *qnrB1* (*n*=4) in *K. pneumoniae* from spinach and water, *qnrS1* (*n*=1) in *E. coli* from spinach and *qnrB6* (*n*=3), *qnrB37* (*n*=5), *qnrE1* (*n*=10) in *S. fonticola* from spinach and water, while *mdtK* (*n*=4), and *mdtH* (*n*=3) were present in *S. fonticola* from water only. The *qnrB17* resistance gene was present in *K. pneumoniae* (*n*=4) and *S. fonticola* (*n*=2) strains from spinach and water (Figure 1). The *S. enterica* strain isolated from irrigation water also harboured *aac(6')-Iaa* and *aac(6')-Iy* aminoglycoside resistance genes (Figure 1) and a *S. fonticola* strain from irrigation water harboured an aminoglycoside [*aph(3")-Ib*] and sulfonomide (*sul2*) resistance gene (Figure 1).

### Detection of Mobile Genetic Elements and Association to Antimicrobial Resistance Genes

Known resistance plasmid replicon types of Enterobacterales including IncFIB, IncFIA, IncFII, IncB/O, and IncHI1B were observed in all strains following analysis with PlasmidFinder (data not shown). The β-lactamase gene, *bla*_CTX-M-15_, was the only resistance gene associated with plasmids (IncFII_pKP91 and/or IncFIB(K)_1_Kpn3) in four *K. pneumoniae* strains upon further analysis (Table 3). The IS6 family elements (IS6100) have been reported to play a pivotal role in the dissemination of resistance determinants in gram-negative bacteria (Partridge et al., 2018), and were observed in relation to the *dfrA14b* resistance gene in all five *K. pneumoniae* strains (Table 3). The *bla*_CTX-M-14_ and *sul2* resistance genes were related to the ISEcp1 element within the IS1380 family in one *E. coli* and three *K. pneumoniae* strains, respectively, while one *S. fonticola* strain carried a *sul2* gene that was related to IS110 (Table 3). One *E. coli* strain carried the *qnrS1* resistance gene that was related to ISKra4. Other insertion sequences detected belonged predominantly to the IS3 and IS110 families (data not shown), with one *K. pneumoniae* strain carrying the *bla*_SHV-80_ broad spectrum β-lactamase that was related to IS3 (Table 3). In all *K. pneumoniae* strains (*n*=5) where the *qnrB1* resistance gene was present, association to Tn5403 was seen (Table 1). In one *E. coli* and five *K. pneumoniae* strains, integron In191 was observed, with *dfrA14* in the cassette array (Table 3).

**Table 3:** Extended-spectrum β-lactamase (ESBL)/AmpC β-lactamase (AmpC)-producing Enterobacterales with resistance genes related to mobile genetic elements.

Isolate information			Resistance genes associated with mobile genetic elements					
			Genes		Mobile genetic elements			
Source	Strain	Species	β-lactamase	Other	Plasmids	Insertion sequence families	Transposons	Integron
W	UPMP2130	*Escherichia coli*	CTX-M-14			IS1380		
S	UPMP2120	*Escherichia coli*		qnrS1		ISKra4		
				dfrA14b				In191
W	UPMP2112	*Klebsiella pneumoniae*	SHV-80			IS3		
			CTX-M-15		IncFIB(K)_1_Kpn3	IS1380		
				sul2				
				qnrB1			Tn5403	
				dfrA14b		IS6		In191
W	UPMP2118	*Klebsiella pneumoniae*	TEM-1B			IS1380		
				dfrA14b		IS6		In191
				qnrB1			Tn5403	
S	UPMP2114	*Klebsiella pneumoniae*	CTX-M-15		IncFII_pKP91			
					IncFIB(K)_1_Kpn3			
				sul2		IS1380		
				qnrB1			Tn5403	
				dfrA14b		IS6		In191
S	UPMP2121	*Klebsiella pneumoniae*	CTX-M-15		IncFII_pKP91			
			TEM-1B			IS1380		
				qnrB1			Tn5403	
				dfrA14b		IS6		In191
S	UPMP2122	*Klebsiella pneumoniae*	CTX-M-15		IncFII_pKP91			
					IncFIB(K)_1_Kpn3			
				sul 2		IS1380		
				qnrB1			Tn5403	
				dfrA14b		IS6		In191
W	UPMP2116	*Serratia fonticola*		sul2		IS110		

W, water; S, spinach.

### *In silico* Analysis of Serotypes, Multi-Locus Sequence Types, and Virulence Factors

The *in silico* MLST analysis, predicted serotypes, and pathogenicity probability of all 19 strains are shown in Table 2. Three different sequence types (ST58, ST117, and ST10) and three different serotypes (O75:H9, O11:H4, and O8:H17) were observed in the three *E. coli* strains. The five *K. pneumoniae* strains belonged to three different sequence types and three different serotypes (KL27, KL24, and KL39) which were observed based on the K-antigen, while the O-serotype included O4 and O1 (Table 2). The predicted antigenic profile of the *S. enterica* strain was O11:k:1,2. Furthermore, the *S. enterica* strain contained 11 *Salmonella* SPI, namely SPI-1, SPI-2, SPI-3, SPI-4, SPI-5, SPI-9, SPI-13, SPI-14, one unnamed, as well as the centisome 63 (C63PI) and 54 (CS54) pathogenicity islands, each harbouring between 20 and 60 virulence factors (Supplementary Table S1). A total of 42 virulence genes were identified in the *E. coli* and *K. pneumoniae* strains (Supplementary Tables S2, S3). Of these, 20 were detected in *E. coli* strains only and 20 in *K. pneumoniae* strains only, while *fyuA* (iron uptake associated with siderophores) and *irp2* (iron uptake) virulence factors were detected in two *E. coli* strains from irrigation water as well as three *K. pneumoniae* strains from spinach samples. All three *E. coli* strains carried the *terC* (tellurite resistance) virulence gene (Supplementary Table S2) and in all five *K. pneumoniae* strains, the *mrkA*, *mrkB*, *mrkC*, *mrkD*, *mrkE*, (main structural subunit and assembly machinery for type 3 fimbriae) *mrkH* (regulatory protein), and *mrkI* (DNA binding protein) virulence factors were present (Supplementary Table S3). No shiga-toxin producing genes were present in the *E. coli* strains. A total of 89 virulence factors were identified in the *S. fonticola* strains (Supplementary Table S4). This included 25, 18, 16, and 6 of the virulence factors present in 100% (*n*=10), 90, 80, and 70% of the selected *S. fonticola* strains, respectively, while the remaining 24 virulence factors were present in varying numbers in 1–6 of the strains (Supplementary Table S4). The *iroN* salmochelin siderophore receptor which plays a role in disease establishment was present in three *S. fonticola* strains (two from unwashed baby spinach samples at the retailer and one from the irrigation pivot point water), one *E. coli* strain from the ground water, as well as in the SPI-13 in the *S. enterica* strain from river irrigation water. Relevant similarities to human pathogens were predicted for all 19 strains with a confidence of 0.635–0.721 in the *S. fonticola* strains (*n*=10), 0.852–0.931 in the *E. coli* strains (*n*=3), 0.796–0.899 in the *K. pneumoniae* strains (*n*=5), and 0.939 in the *S. enterica* strain (Table 2).

## Discussion

To the authors knowledge, this is the first study to use WGS for in-depth molecular characterization of ESBL/AmpC-producing *E. coli*, *K. pneumoniae*, *S. enterica*, and *S. fonticola* isolates, previously identified and partially characterized, from spinach and irrigation water samples in commercial production chains (Richter et al., 2020). Characterization included AMR, mobile genetic elements (e.g., insertion sequences, plasmids, and integrons), serotypes, and determining the pathogenicity. All these factors are crucial in defining and attributing infection sources of food-related outbreaks caused by resistant microorganisms (Oniciuc et al., 2018). Overall, the results corresponded with main global findings where AMR genes and associated mobile genetic elements have been reported in Enterobacterales from fresh produce and irrigation water, with the potential to pose a health risk to humans upon exposure (Jones-Dias et al., 2016; Finton et al., 2020). Previously, the presence of *intI3* was reported in a high percentage of isolates from the current study following conventional PCR and sequencing (Richter et al., 2020). However, in-depth WGS analysis showed that no *attI* fragment preceded the *IntI3* genes; consequently, the *IntI3* genes detected did not form part of complete integrons, which typically include an integrase *intI* gene encoding a site-specific recombinase, a recombination site *attI* as well as a promoter (P_c_; Kaushik et al., 2018). Overall, six isolates in the current study were positive for Class 1 integrons (In191), similar to In191 positive clinical ESBL-producing Enterobacterales from an academic teaching hospital in Pretoria, SA (Sekyere et al., 2020). Additionally, these MDR environmental isolates harbored various virulence factors central to pathogenicity, including genes associated with urinary tract infections and iron sequestering systems crucial for disease establishment. All isolates had relevant similarity to human pathogens and form part of the WHO 3rd generation cephalosporin resistant critical priority pathogens (WHO, 2017).

Two of the *E. coli* strains from the current study harboured plasmid-mediated AmpC *bla*_CMY-2-like_ genes (*bla*_CMY-113_ and *bla*_CMY-101_), which correspond to the phenotypic profile of resistance to expanded-spectrum cephalosporins previously reported for these isolates using traditional PCR analysis (Richter et al., 2020). The *bla*_CMY-2_ pAmpC genes are the most commonly reported in *E. coli* and other Enterobacterales species and have clinical relevance, as it inactivates third generation cephalosporins and mediates resistance to carbapenems (Jacoby, 2009; Bortolaia et al., 2014). Three different multi-locus sequence types, namely ST58, ST10, and ST117, were identified in the *E. coli* isolates. Isolated from the retailed unwashed spinach samples in the current study, ST58 *E. coli* have previously also been associated with human extra-intestinal infections including sepsis, and have emerged worldwide in wild and food-production animals (Reid et al., 2020). As an example, ST58 *E. coli* with serotype O75:H9 corresponded to an *E. coli* strain of bovine origin from Pakistan and also carried the IncFIB plasmid (Ali et al., 2020). Although the strain from the current study had less AMR genes than reported in ST58 *E. coli* with serotype O75:H9 by Ali et al. (2020), the trimethoprim (*dfrA14*), fluoroquinolone (*qnrS1*), and β-lactam (*bla*_CTX-M-15_) genes corresponded. Similarly, uropathogenic ST58 *E. coli* with resistance to fluoroquinolone and trimethoprim have previously been isolated from hospital patients in Australia (McKinnon et al., 2018). The *bla*_CTX-M-15_ gene identified in the ST58 *E. coli* strain from the current study was associated with the ISKra4 insertion sequence, previously identified in *K. pneumoniae* harbouring *bla*_CTX-M-15_, and was responsible for the movement to different parts of the genome through a replicative transposition mechanism (Razavi et al., 2020). In contrast to Hauser et al. (2013) who identified food-associated shiga-toxin producing *E. coli* ST58, no *stx* genes were present in the strains. The *E. coli* ST58 from the current study harboured the *gad* (glutamate decarboxylase) virulence gene, similar to *E. coli* ST58 strains isolated from aragula (rocket; Reid et al., 2020). However, the presence of *lpfA* (long polar fimbriae) and *terC* (tellurium ion resistance protein) virulence factors in the strain from the current study contrasted the virulence gene profiles reported by Reid et al. (2020). *Escherichia coli* ST10 have previously been associated with human clinical infections and has been isolated from different sources including recreational and/or wastewater samples (Falgenhauer et al., 2019). From the current study, the *E. coli* ST10 with serotype O8:H17 was isolated from borehole water used for irrigation. Although this sequence type has previously been associated with shiga-toxin-producing *E. coli* (STEC; Gonzalez-Escalona and Kase, 2018), no *stx* genes were detected in the current study. The virulence factors present were *terC* (tellurium ion resistance protein), *astA* (EAST-1 heat-stable toxin), *fyuA* (ferric yersiniabactin uptake receptor), *irp2* (nonribosomal peptide synthetases), *iss* (increased serum survival), and *sitA* (iron transport protein). Previously, *E. coli* ST10 with similar virulence gene profiles were isolated from human blood cultures and reported as extra-intestinal pathogenic *E. coli* (ExPEC; Maluta et al., 2017). Additionally, ESBL-producing *E. coli* ST10 of the same serotype have been isolated from wastewater and are depicted as a probable environmental reservoir of *bla*_CTX-M_ genetic determinants (Tanaka et al., 2019). In the current study, the ST58 *E. coli* strain harboured the *bla*_CTX-M-15_ genetic determinant, while *bla*_CTX-M-14_ was present in the ST10 *E. coli* strain. Globally, the CTX-M type ESBLs (especially *bla*_CTX-M-14_ and *bla*_CTX-M-15_) have become the dominant genotype and the most widely distributed (Cantón et al., 2012; Adamski et al., 2015). *Escherichia coli bla*_CTX-M-14_ positive strains have previously been isolated from store-bought produce in Germany and South Africa (Richter et al., 2019; Reid et al., 2020), food producing animals in China (Liao et al., 2015) and clinical settings in Brazil and South Africa (Cergole-Novella et al., 2010; Peirano et al., 2011).

The third *E. coli* sequence type (ST117) detected from irrigation source water in the current study has previously been reported as part of a group of multi-serotype extra-intestinal pathogenic *E. coli* (ExPEC) and avian pathogenic *E. coli* (APEC) strains (Kim et al., 2017). The *E. coli* ST117 strain from the current study harboured 20 virulence factors including the ExPEC *hlyF* (Hemolysin F) virulence gene. In previous studies, *stx* genes were identified in *E. coli* strains with the same STs detected in the current study, yet the virulence gene content and serotypes differ from the strains in the current study (Gonzalez-Escalona and Kase, 2018). However, the three non-STEC *E. coli* strains (ST58, ST10, and ST117) from the current study had a 93, 89, and 85% probability of being human pathogens, based on the pathogenic protein families.

In addition to *E. coli*, other Enterobacterales isolates harbouring *bla*_CTX-M-15_ have also been detected in different environments. In the current study, all five *K. pneumoniae* strains harboured the *bla*_CTX-M-15_ genetic determinant. The prevalence and dissemination of *bla*_CTX-M_ throughout various environments globally underlines the different contamination routes through which fresh produce may also become contaminated with these MDR organisms. For instance, Gekenidis et al. (2020) have demonstrated the long-term persistence of *E. coli* harbouring *bla*_CTX-M-15_ in soil and lettuce after its introduction *via* irrigation water. Similarly, *bla*_CTX-M-15_ positive ST985 *K. pneumoniae* strains were present in spinach at harvest on the farm as well as retail samples after processing in the current study, suggesting successful persistence of these MDR strains. In four *K. pneumoniae* strains (ST3559, *n*=1 and ST985, *n*=3), the *bla*_CTX-M-15_ genes were associated with IncF replicons (IncFII_K_ and IncFIB) which have previously been linked to diverse *K*. *pneumoniae* outbreak strains (Dolejska et al., 2012, 2013; Löhr et al., 2015). Moreover, in *K. pneumoniae* ST3559, *bla*_CTX-M-15_ was also associated with *ISEcp1* (also called *ISEc9*), a member of the widely reported IS1380 family, and can enable the independent transposition with insertion mutation and genetic relocations (Partridge, 2011). The *K. pneumoniae* strains in the current study also harboured *bla*_SHV_ ESBL encoding genes (*bla*_SHV-187_, *bla*_SHV-106_, and *bla*_SHV-178_). Previously, SHV genetic determinants were reported in *K. pneumoniae* from hospitals and receiving wastewater treatment plants in Romania (Surleac et al., 2020) as well as irrigation water and agricultural soil in South Africa (Iwu et al., 2020; Richter et al., 2020). Interestingly, the *K. pneumoniae* ST15 strain isolated from water in the current study harboured *bla*_SHV-106_ which Liakopoulos et al. (2016) previously reported to be geographically constrained and have only been described in *K. pneumoniae* isolates from Portugal together with *bla*_TEM-1_. Similarly, the *K. pneumoniae* ST15 strain from the current study also harboured *bla*_SHV-106_ together with *bla*_TEM-1_. *Klebsiella pneumoniae* ST15 is regarded as an emerging international high-risk clone causing nosocomial outbreaks worldwide with high-levels of antibiotic resistance including production of ESBLs, mainly CTX-M-15 (Han et al., 2021).

The *K. pneumoniae* ST3559 strain isolated from irrigation water in the current study was capsular type 27 and serotype O4, which is similar to an O4 serotype MDR *K. pneumoniae* outbreak strain from a neonatal care unit in sub-Saharan Africa (Cornick et al., 2020). In addition, *K. pneumoniae* ST3559 harboured the *bla*_SHV-178_ gene which, to the best of our knowledge, have previously only been reported in clinical *Enterobacter hormaechei* strains from the First Affiliated Hospital of Zhejiang University in Hangzhou (Gou et al., 2020). Apart from β-lactamase genes, the *K. pneumoniae* strains also harboured aminoglycoside, fosfomycin, fluoroquinolone, tetracyline, phenicol, trimethoprim, and sulfonomide resistance genes, which is a greater diversity of resistance genes than previously reported in Enterobacterales isolates from German surface waters (Falgenhauer et al., 2019). Similar to the results of clinical *K. pneumoniae* strains reported by Mbelle et al. (2020), In191, harbouring *dfrA14* was identified in the three different *K. pneumoniae* sequence types of the current study, reiterating that it is not a narrow spectrum integron. In addition, *dfrA14b* was associated with *IS6* that has previously been reported as having a vital role in the rearrangement and dissemination of antibiotic resistance (Varani et al., 2021). The presence of *fosA* and *sul2* in all the *K. pneumoniae* strains of the current study also corresponds to the results reported by Mbelle et al. (2020) from clinical *K. pneumoniae* strains in Pretoria. The high-level of trimethoprim resistance globally has however led to trimethoprim-sulfamethoxazole no longer being recommended for outpatient treatment of urinary tract infections and similarly, the use of fosfomycin might not be efficacious anymore (Mbelle et al., 2020). Four MDR *K. pneumoniae* isolates from irrigation water (ST15, *n*=1) and spinach (ST985, *n*=3) had O1 serotypes, previously reported as the most commonly isolated serotypes from human hosts and dominant in human disease (Follador et al., 2016). However, it is noteworthy that no genes encoding carbapenamases nor resistance to colistin were identified in the current study. All five characterized *K. pneumoniae* strains also harbored several virulence factors including those that coded for an iron uptake system (*kfu*) and type 3 fimbrial adhesins (*mrk*) that play an important role in adhesion to medical devices such as catheters (Albasha et al., 2020; Finton et al., 2020).

*Serratia* spp. are opportunistic pathogens that may pose a health threat to immunocompromised and hospitalized patients (Petersen and Tisa, 2013). The *S. marcescens* species is most often associated with nosocomial infections; however, *S. fonticola* has been reported to function as a human pathogen when detected alone or may be a bystander and act as a carrier of resistance genes when discovered with other organisms (Petersen and Tisa, 2013; Aljorayid et al., 2016). Characterizing virulence genes of the MDR environmental strains therefore becomes important within the plant-food producing environment. In the current study, all *S. fonticola* strains harboured *bla*_SFO-1_ and numerous plasmid incompatibility (Inc) groups were identified in these *S. fonticola* strains (data not shown). However more in-depth plasmid typing and analysis will be required to fully understand the risk/probability of *bla*_SFO-1_ dissemination in the environment where *S. fonticola* naturally occurs. In certain Enterobacterales species, ESBL genes are inherently carried on chromosomes (Naas et al., 2008). This includes the *bla*_SFO-1_ ESBL gene from *S. fonticola* that differs from most class A ESBLs, as the β-lactamases’ production can be induced by a high level of imipenem (Naas et al., 2008). The *bla*_SFO-1_ ESBL does not form part of the most clinically relevant ESBLs and are therefore rarely reported. Zhou et al. (2020) reported in contrast an increasing trend of the co-existence of plasmid-borne *bla*_SFO-1_ and carbapenemase genes in clinical *Enterobacter* spp. in China. All the *S. fonticola* strains also harboured numerous fluoroquinolone resistance genes, raising a health concern for treatment options, as fluoroquinolones are often used for management of conditions including typhoid fever and MDR tuberculosis (Richards et al., 2019). Interestingly, one *S. fonticola* strain harboured an acquired trimethoprim (*sul2*) resistance gene associated with IS110, corresponding to *K. pneumoniae* from a German university hospital (Schwanbeck et al., 2021). The *Serratia* genus naturally lacks resistance genes for trimethoprim and sulfonamides (Sandner-Miranda et al., 2018). Previous reports of potential pathogenic *S. fonticola* primarily focused on the antibiotic resistance profiles (Tasić et al., 2013; Aljorayid et al., 2016; Hai et al., 2020). The strains from the current study additionally harboured various virulence factors. This included flagellar biosynthesis- and chemotaxis-related genes as well as genes encoding iron uptake systems corresponding to those previously reported in important MDR nosocomial pathogenic *S. marcescens* (Iguchi et al., 2014).

Only one *S. enterica* strain isolated from river irrigation water was characterized in the current study. Irrigation water is well documented as a source for fresh produce contamination of foodborne pathogens including *Salmonella* spp. (Liu et al., 2018). The strain harboured an AmpC resistance gene, similar to *S. enterica* characterized from surface water in the United States (Li et al., 2014). In addition, the *S. enterica* from the current study carried aminoglycoside resistance genes [*aac(6')-Iaa* and *aac(6')-Iy*], similar to results reported by Nair et al. (2016) for non-typhoidal *Salmonella* spp. isolated from a United Kingdom population. Of the 23 known *Salmonella* SPIs previously described (Mansour et al., 2020), the isolate from the current study carried 11 SPIs. This included SPIs that are commonly reported in *S. enterica* and encode genes responsible for enabling invasion of epithelial cells (SPI1), facilitating the replication of intracellular bacteria (SPI2), adhesion to epithelial cells (SPI3, 4, 5, and 9; Waterman and Holden, 2003; Velásquez et al., 2016; Mansour et al., 2020), as well as SPI13 and 14 which corresponds to being part of the core genome of invasive non-typhoidal *Salmonella* spp. (Suez et al., 2013). Additionally, pathogenicity islands C63PI and CS54 were present in the *S. enterica* strain in this study, which has previously been found in the S. Typhimurium and S. Typhi genomes (Sabbagh et al., 2010; Jibril et al., 2021). Since no phenotypic indication of virulence was investigated, the prediction of virulence genes using *in silico* tools should be regarded with care; however, using PathogenFinder, the *S. enterica* strain from the current study showed 94% probability of being a human pathogen.

## Conclusion

This is the first WGS analysis study of MDR ESBL/AmpC-producing *E. coli*, *K. pneumoniae*, *S. fonticola*, and *S. enterica* isolates from spinach production systems within South Africa. The selected isolates represent potential pathogenic genera listed by the WHO as a priority for surveillance of AMR screening. Numerous clinically relevant resistance genes were detected in the screened samples. This study showed the potential of using WGS in metadata studies for detailed molecular characterization of potential pathogenic Enterobacterales. Furthermore, the study highlighted the importance of the agricultural production environment as a source of antibiotic resistance genes within Enterobacterales at the water-plant-food interface. A more in-depth and controlled analysis, with a greater number of sequenced isolates from the farm-to-retail supply chain is required to better understand the prevalence and resistance gene transmission through the supply chain. The results from this study further highlight the need for expanded surveillance in agricultural systems.

## Data Availability Statement

The nucleotide sequences of the 19 Enterobacteriaceae strains described in this paper were deposited in the National Center for Biotechnology Information GenBank database in the BioProject number: PRJNA642017, accession numbers NZ_JACAAL010000000, NZ_JACBIV000000000-NZ_JACBJE000000000, and NZ_JACNYM000000000-NZ_JACNYT000000000 (Table 2).

## Author Contributions

EP, SD, LR, and LK contributed to the conception and design of the study. LR performed the experiments. LR, SD, MA, and AI analyzed the data. LR, EP, and SD contributed to interpretation and presentation. SD, EP, and LK were involved in funding acquisition. All authors contributed to the article and approved the submitted version.

## Funding

This research was funded by the Water Research Commission (WRC) funded project “Measurement of water pollution determining the sources and changes of microbial contamination and impact on food safety from farming to retail level for fresh vegetables” (WRC Project No K5/2706/4 and WRC Knowledge Review 2017/18) and the Partnerships for Enhanced Engagement in Research (PEER) a USAID/DST funded project “Characterizing and tracking of antimicrobial resistance in the water-plant-food public health interface” (Grant no. 48). Furthermore, the financial assistance of the Department of Science and Innovation (DSI) – National Research Foundation (NRF) Centre of Excellence in Food Security funded this research under the Food Safety Program’s “Safe Food for the Food Insecure” project (Project 160301 and 160302). This work is based on the research supported in part by the NRF of South Africa [Grant specific unique reference number (UID): 74426 and Grant Number: 120319]. Opinions expressed and conclusions arrived at, are those of the authors and are not necessarily to be attributed to the NRF.

## Conflict of Interest

The authors declare that the research was conducted in the absence of any commercial or financial relationships that could be construed as a potential conflict of interest.

## Publisher’s Note

All claims expressed in this article are solely those of the authors and do not necessarily represent those of their affiliated organizations, or those of the publisher, the editors and the reviewers. Any product that may be evaluated in this article, or claim that may be made by its manufacturer, is not guaranteed or endorsed by the publisher.

## Footnotes

^1^ https://digitalinsights.qiagen.com

^2^ https://pubmed.ncbi.nlm.nih.gov/27342282/

^3^ https://github.com/tseemann/abricate

^4^ https://cge.cbs.dtu.dk/services/

^5^ https://bigsdb.pasteur.fr/klebsiella/klebsiella.html

^6^ http://integrall.bio.ua.pt
